# *Plasmodium vivax* Cell Traversal Protein for Ookinetes and Sporozoites (CelTOS) Functionally Restricted Regions Are Involved in Specific Host-Pathogen Interactions

**DOI:** 10.3389/fcimb.2020.00119

**Published:** 2020-03-24

**Authors:** Gabriela Arévalo-Pinzón, Diego Garzón-Ospina, Fredy A. Pulido, Maritza Bermúdez, Johanna Forero-Rodríguez, Xandy M. Rodríguez-Mesa, Leidy P. Reyes-Guarín, Carlos F. Suárez, Manuel A. Patarroyo

**Affiliations:** ^1^Receptor-Ligand Department, Fundacion Instituto de Inmunologia de Colombia (FIDIC), Bogota, Colombia; ^2^School of Medicine and Health Sciences, Universidad del Rosario, Bogota, Colombia; ^3^PhD Programme in Biomedical and Biological Sciences, Universidad del Rosario, Bogota, Colombia; ^4^Molecular Biology and Immunology Department, Fundacion Instituto de Immunologia de Colombia (FIDIC), Bogota, Colombia; ^5^Biomathematics Department, Fundacion Instituto de Immunologia de Colombia (FIDIC), Bogota, Colombia

**Keywords:** malaria, multi-epitope multi-stage vaccine, sporozoite, *P. vivax*, CelTOS

## Abstract

Following the injection of *Plasmodium* sporozoites by a female *Anopheles* mosquito into the dermis, they become engaged on a long journey to hepatic tissue where they must migrate through different types of cell to become established in parasitophorous vacuoles in hepatocytes. Studies have shown that proteins such as cell traversal protein for *Plasmodium* ookinetes and sporozoites (CelTOS) play a crucial role in cell-traversal ability. Although CelTOS has been extensively studied in various species and included in pre-clinical assays it remains unknown which *P. vivax* CelTOS (*Pv*CelTOS) regions are key in its interaction with traversed or target cells (Kupffer or hepatocytes) and what type of pressure, association and polymorphism these important regions could have to improve their candidacy as important vaccine antigens. This work has described producing a recombinant *Pv*CelTOS which was recognized by ~30% *P. vivax*-infected individuals, thereby confirming its ability for inducing a natural immune response. *Pv*CelTOS' genetic diversity in Colombia and its ability to interact with HeLa (traversal cell) and/or HepG2 cell (target cell) external membrane have been assessed. One region in the *Pv*CelTOS amino-terminal region and another in its C-terminus were seen to be participating in host-pathogen interactions. These regions had important functional constraint signals (ω < 0.3 and several sites under negative selection) and were able to inhibit specific r*Pv*CelTOS binding to HeLa cells. This led to suggesting that sequences between aa 41–60 (40833) and 141–160 (40838) represent promising candidates for an anti-*P. vivax* subunit-based vaccine.

## Introduction

*Plasmodium vivax* is the second most important species causing human malaria, putting around 35% of the world's population at risk of infection (Howes et al., 2016). However, because infections caused by this species are rarely lethal, there are few studies related to controlling and eliminating this species compared to that reported for other species such as *Plasmodium falciparum*. It is worth highlighting that in spite of its low parasitaemia levels, *P. vivax* clinical infection can be debilitating, producing significant economic and health-related impacts in many countries where it is predominant. *P. vivax*'s biological and epidemiological characteristics impose significant challenges regarding the search for treatment and its biological study (Mueller et al., 2009; Howes et al., 2016). Such characteristics would include its ability to remain in latent hypnozoite form during hepatic stage (leading to repeated relapses and clinical attacks) (White and Imwong, 2012), its high transmission potential caused by high and early gametocyte production, high infectivity in mosquitos and its short development cycle in the vector (Mueller et al., 2009); these have imposed important challenges for designing effective antimalarial treatments and vaccines for this species.

One of the main obstacles when designing an anti-malarial vaccine is the high polymorphism within the main proteins expressed during *Plasmodium* sporozoite (Spz) and merozoite (Mrz) invasion and the alternate use of invasion routes (Lopaticki et al., 2011; Cornejo et al., 2014). These two evasion mechanisms (polymorphism and alternative invasion routes) make it necessary to evaluate alternatives for covering the many variants and diversity of proteins used during invasion. Genetic diversity and evolutionary force analysis (drift, selection, and recombination) are useful tools for reducing the amount of candidates to be studied to advance this search.

Recent studies of *P. vivax* proteins have shown that Spz genes are more conserved than the Mrz genes studied to date (Garzon-Ospina et al., 2018). Analyzing the main putative domains or regions of Spz proteins involved in cell traversal, gliding motility, and hepatocyte binding has highlighted negative selection signals probably responsible for the studied proteins' low diversity. This type of selection probably maintains the integrity of the proteins' functional regions. Such evolutionary patterns have been found in *Pv*P52, *Pv*P36, *P. vivax* secreted protein with altered thrombospondin repeat domain (*Pv*SPATR), *P. vivax* perforin-like protein 1 (*Pv*PLP1), *P. vivax* merozoite capping protein 1 (*Pv*MCP1), *P. vivax* thioredoxin-like protein (*Pv*TLP), *P. vivax* cell traversal protein for ookinetes and sporozoites (*Pv*CelTOS), and *Pv*MB2 (Garzon-Ospina et al., 2018). This makes them strong antigen candidates for in-depth study for a vaccine against *P. vivax*.

CelTOS is a promising candidate; it is expressed in *Plasmodium* ookinete and Spz micronemes. This protein's importance became evident in studies of *Plasmodium berghei celtos* genetic disruption; it was found that the absence of this gene reduced Spz infectivity in the liver and significantly inhibited cell traversal by ookinetes in mosquito midgut wall (vector cells) and Spz traversal of different kinds of cells (Kariu et al., 2006). Using a promoter-swapping technique in *P. yoelii* enabled measuring CelTOS participation in infection with greater certainty. Such experiments led to finding that even though CelTOS is not essential for successful hepatocyte infection *in vitro*, it clearly has a function in Spz cell traversal (Steel et al., 2018). *Py*CelTOS participation in oocyst survival/development and Spz gliding motility has been shown (Steel et al., 2018), having significant implications for including it in an anti-malarial vaccine since antibodies (Abs) targeting this protein would be able to block more than one process during pre-erythrocyte stage.

It has been found that *Pv*CelTOS 3D structure resembles that of proteins binding to and disrupting membranes; this has been sufficient to support the idea of *Pf* CelTOS and *Pv*CelTOS binding to phosphatidic acid on plasma membranes' inner leaflet (Jimah et al., 2016) thereby facilitating parasite exit during cell traversal. However, immunological and functional analysis has shown that Abs targeting CelTOS strongly inhibit gliding motility, cell traversal, Spz hepatocyte infection and impair the parasite's development in a mosquito vector, suggesting that it has a key role in early events during the pre-erythrocyte stage regarding traversed or target cells (Kupffer or hepatocytes) (Bergmann-Leitner et al., 2010; Espinosa et al., 2017). Immunological data has shown that *Pf* CelTOS-derived peptides were capable of stimulating PBMCs isolated from volunteers immunized with irradiated Spz. Such response was correlated with better protection in volunteers, thereby demonstrating CelTOS' potential as vaccine candidate (Doolan et al., 2003). Studies have been focused on evaluating this protein's antigenic, immunogenic, and functional potential based on these results.

*Pf* CelTOS immunization studies in mice have shown its ability for inducing sterile protection against a heterologous challenge with *P. berghei* Spz in both inbred and outbred mice (Bergmann-Leitner et al., 2010). Therefore, mice immunized with recombinant *P. falciparum* CelTOS in combination with glucopyranosyl lipid adjuvant-stable emulsion (GLA-SE) or glucopyranosyl lipid adjuvant-liposome-QS21 (GLA-LSQ) adjuvant system have significantly inhibited infection by the chimeric *P. berghei* parasite expressing *Pf* CelTOS (Espinosa et al., 2017). *Pb*CelTOS formulation in a new adjuvant system named gene-mediated inactivated vaccine (GeMI-Vax) platform in *Shigella* was able to induce protection against *P. berghei* Spz challenge (Bergmann-Leitner et al., 2013). Patients naturally infected by *P. vivax* contain Abs showing that *Pv*CelTOS is antigenic, predominantly toward the carboxyl terminal region (Rodrigues-Da-Silva et al., 2017).

Based on CelTOS' potential as vaccine candidate and functional evidence regarding critical processes during *Plasmodium* invasion, this investigation has been focused on broadening and analyzing information about *Pv*CelTOS' genetic diversity obtained from two of Colombia's endemic regions. This protein's antigenicity was also evaluated using samples from endemic areas and the *Pv*CelTOS region responsible for specific interaction with receptors on HeLa cells (used as cell-traversal model) was ascertained by highly robust and specific binding assays (FACS and radiolabelled). Comparing results of genetic diversity and host cell binding studies revealed that regions having functional constraint signals were the regions directly participating in host cell interaction, suggesting that *Pv*CelTOS contains key conserved regions which could be useful for designing control methods targeting *P. vivax* strains. These results also emphasized *Pv*CelTOS as an antigen worth considering when designing a vaccine against *P. vivax* due to its exposure to a host's immune system, its low genetic diversity and being involved in host-pathogen interactions.

## Materials and Methods

### Declaration of Ethics and Information Regarding Samples

The blood samples were collected from patients who were attending health centers during 2010 to 2015 due to having symptoms of the disease. Peripheral blood samples from patients diagnosed with *P. vivax* malaria by microscopic examination were collected and placed in EDTA-containing Vacutainer tubes. Three hundred microliter of peripheral blood samples were then processed to extract total DNA using a Wizard Genomic DNA Purification kit (Promega), following the manufacturer's recommendations. *Plasmodium vivax* genomic DNA (gDNA) was confirmed by small ribosomal subunit PCR amplification. Sixty-two samples were used for polymorphism analysis, 46 from Chocó and 16 from Córdoba, these being two Colombian regions having very high transmission rates. Serum samples (*n* = 32) from endemic regions in Colombia were used for antigenicity studies. Ten naïve individuals living in Bogotá (non-endemic region) and having had no reported malarial episodes were used as control subjects during antigenicity studies.

All patients who provided blood samples were informed about the study's objective; they then signed an informed consent form prior to blood collection. All patients enrolled in the study were adults and treated according to Colombian health policy. The procedures involved in sample taking had been approved by Fundación Instituto de Inmunología de Colombia (FIDIC) (IRB: ACTA N° 037-CEEPA) and the Universidad del Rosario's ethics committees (IRB: CEI-ABN026-0001061).

### Primer Design and PCR *pvceltos* Amplification

The Sal-I reference strain's genome region (PlasmoDB access number: Pv_Sal1_chr14:1480489–1482488) containing the *pvceltos* gene was used for designing two sets of primers. The first set (*pvceltosGVfw* 5′-GCATCCCATTGTACAAGCAC-3′ and *pvceltosGVrev* 5′-TATGCATATAACTTTTTCTC-3′) was designed for evaluating *pvceltos* genetic diversity in two of Colombia's endemic populations (Chocó *n* = 46 and Córdoba *n* = 16). The second set (*pvceltosRPfw 5*′ATGAACAAAGTAAACCGAGTCTCG3′ and *pvceltosRPrev 5*′CTCATCAGAGAACTCATCTTCAGCTTC3′) was designed for obtaining a recombinant *Pv*CelTOS (r*Pv*CelTOS) fragment comprising 12–196 aa residues from the 196-residue full-length polypeptide (PlasmoDB accession number: PVX_123510). A KAPA HiFi PCR kit was used for amplifications at 25 μL final volume [7.5 μL nuclease-free water, 12.5 μL ReadyMix, 1.5 μL of each primer (5 μM), and 10–40 ng gDNA]. The following thermal profile was used: 1 initial step at 95°C (5 min), 35 cycles at 98°C (20 s), 58°C (30 s) and 72°C (45 s), and a final extension step at 72°C (10 min). A Wizard SV Gel kit and PCR Clean-Up System (Promega) were used for purifying amplification products, following the manufacturers' indications. The sequences were obtained by bidirectionally sequencing using the BigDye method with capillary electrophoresis using an Applied Biosystems' ABI-3730 XL genetic analyser (Macrogen, Seoul, South Korea).

### Analyzing Genetic Diversity, Neutrality, and Natural Selection

CLC Genomics Workbench v.3 software (QIAGEN) was used for analyzing and assembling the electropherograms obtained from sequencing. The Colombian consensus sequences were then aligned with the reference sequences (Carlton et al., 2008; Neafsey et al., 2012; Auburn et al., 2016) (GenBank access numbers: AFMK01001414.1, AFNI01001279.1, AFBK01001982.1, AFNJ01001964.1, and PlasmoDB: PVX_123510) available in PlasmoDB and sequences from an Iranian population (Mehrizi et al., 2017) using MUSCLE alignment software (Edgar, 2004).

DnaSP v.5 software (Librado and Rozas, 2009) was used for evaluating genetic polymorphism by calculating different diversity estimators. These included the amount of polymorphic sites segregating into the target population (Ss), the amount of parsimony-informative sites (Ps, sites having a minimum of two nucleotides occurring at least twice), and singleton sites (S, non-informative sites), the amount of haplotypes analogous to the amount of alleles in the target population, nucleotide diversity [π–the average amount of nucleotide differences between two sequences per site (π is equivalent to heterozygosity)] and Watterson estimator (θ^w^) (the amount of segregating sites per nucleotide site).

Tajima's D (Tajima, 1989), Fu & Li's D^*^ and F^*^ (Fu and Li, 1993) and Fay & Wu's H tests (Fay and Wu, 2000) were computed for testing the hypothesis that all mutations were selectively neutral (Kimura, 1983). The D test is based on the differences between the amount of segregating sites and the average amount of nucleotide differences. The D^*^ test is based on the differences between the total amount of mutations on external genealogy branches and the total amount of mutations. The F^*^ test is based on the differences between the total amount of mutations on external genealogy branches and the average amount of nucleotide differences between pairs of sequences. D^*^ and F^*^ are denoted with an ^*^ since both estimators were computed without an outgroup. Under neutrality, D, D^*^ and/or F^*^ values should be equal to zero whilst values >0 could have been due to balancing selection or genetic drift (regarding D, this could have been the result of population structure) whilst <0 values indicated directional selection or population increase.

The H test is based on the differences between the average amount of nucleotide differences between pairs of sequences and θH, an estimator based on the frequency of the derived variants. Significant negative H values indicated selective sweep. Fu's Fs test is based on haplotype (gene) frequency distribution (Fu, 1997). This test is more sensitive to demographic population expansion. Coalescent simulation was used for obtaining confidence intervals (Librado and Rozas, 2009). Sites having gaps were not taken into account.

The modified Nei-Gojobori method (Zhang et al., 1998) was used for calculating the difference between the average amount of non-synonymous substitutions per non-synonymous site (d_N_) and the average amount of synonymous substitutions per synonymous site (d_S_), with the complete deletion option. The MEGA v.5 *Z*-test (Tamura et al., 2011) was used for determining statistical differences between these rates. The modified Nei-Gojobori method and Jukes-Cantor correction (Jukes and Cantor, 1969) were then used for calculating the difference between the average amount of non-synonymous divergences per non-synonymous site (K_N_) and the average amount of synonymous divergences per synonymous site (K_S_) using *P. vivax* and *P. cynomolgi* sequences (GenBank access number: BAEJ01001557) as a single data set.

A sliding window for omega (ω) rate was constructed for *P vivax* (ω = d_N_/d_S_, intraspecies data) and for *P. vivax*/*P. cynomolgi* (ω = K_N_/K_S_, interspecies data) to assess how evolutionary ω rate varied in the *celtos* gene which could have been an indicator of natural selection action regarding specific *celtos* regions. SLAC, FEL, REL (Kosakovsky Pond and Frost, 2005), IFEL (Kosakovsky Pond et al., 2006), MEME (Murrell et al., 2012), and FUBAR (Murrell et al., 2013) methods were used to identify codon sites under positive or negative selection by estimating the d_S_ and d_N_ rates for each site in the alignment [after identifying recombination points by the GARD method (Kosakovsky Pond et al., 2006)]. Furthermore, McDonald-Kreitman tests (MK) Mcdonald and Kreitman, 1991; Egea et al., 2008) were also used for evaluating natural selection signals, taking Jukes-Cantor correction into account (Jukes and Cantor, 1969). MK test is based on comparing polymorphism (intraspecies data, the polymorphism found in *P. vivax*) to divergence (interspecies data, the divergence between *P. vivax* and *P. cynomolgi*).

Network v.5 (Bandelt et al., 1999) was used for constructing a median-joining haplotype network for determining mutational routes from the observed haplotypes. Arlequin v.3.1 software (Excoffier et al., 2007) was used for estimating the degree of genetic differentiation between populations, using Wright's F-statistics (F_ST_–fixation index).

### Cloning and Sequencing *pvceltos* Fragments for Expressing Them

Purified PCR products for obtaining the *pvceltos* fragment were ligated into pEXP5-CT/TOPO vector in frame with a His tag at the carboxyl terminal extreme to enable their purification by affinity chromatography and their identification by Western blot, flow cytometry and IFA studies, using anti-polyhistidine Abs. The ligation product was used for transforming *E. coli* JM109 cells (Promega), following the manufacturer's indications. Recombinant clones were then confirmed by PCR; an UltraClean 6 Minute Mini Plasmid Prep kit (MO BIO) was used for purifying the plasmids and correct insert orientation was evaluated by sequencing, using universal primers.

### Expressing and Obtaining r*Pv*CelTOS

Recombinant pEXP5-*Pv*CelTOS plasmid was used for transforming BL21-AI strain *E. coli* cells (Invitrogen), following the manufacturer's instructions. Transformed cells were cultured in Luria-Bertani (LB) broth with 100 μg/mL ampicillin and 0.1% D-glucose at 37°C overnight using a Labline Orbital Incubator Shaker. The initial inoculum was sown in 1L LB in the aforementioned conditions at 37°C until reaching 0.5 OD600 optical density. Protein expression was induced by adding 0.2% L-arabinose and incubating at 37°C for 4 h at 250 rpm. The culture was spun at 4,500 rpm for 20 min; the pellet was then suspended in solubilising buffer (Moreno-Perez et al., 2017), lysozyme and a protease inhibitor cocktail (Thermo Fisher), as previously described. This mixture was incubated overnight at 10 rpm using a tube rotator (Fisher Scientific, Waltham, USA). Cells were submitted to three thermal shocks to increase cell rupture. The supernatant was collected by spinning at 16,000 rpm for 1 h.

The protein was then purified by affinity chromatography which required Ni-NTA agarose resin (QIAGEN). Coomassie blue stained polyacrylamide gel and Western blot were used to confirm that the protein had been obtained. A Micro BCA Protein Assay kit and MultiSkan Go spectrophotometer (Thermo Scientific) were then used for quantification, thereby determining protein concentration.

### *Pv*CelTOS Peptide Chemical Synthesis

The Sal-1 strain (PVX_123510) *Pv*CelTOS sequence was synthesized in sequential 20 non-overlapping amino acid (aa) peptides following the t-Boc solid-phase multiple peptide synthesis strategy. Peptides were cleaved by the low–high hydrogen fluoride technique; reversed-phase high-performance liquid chromatography (RP-HPLC) was used for purification and matrix-assisted laser desorption/ionization time-of-flight mass spectrometry (MALDI-TOF) for assessing peptide identity.

### Enzyme-Linked Immunosorbent Assay (ELISA)

Recombinant *Pv*CelTOS antigenicity and conformation were ascertained by ELISA. Briefly, 2 μg soluble or denatured (at 80°C for 30 min) r*Pv*CelTOS was then immobilized on 96-well-plates overnight at 4°C. Protein which was not bound to the wells was removed by three washes with 0.05% PBS-Tween 20. The plates were incubated with blocking solution containing 5% skimmed milk in 0.05% PBS-Tween for 1 h. Serum samples from *P. vivax*-infected Colombian individuals (*n* = 32) or malaria-naive individuals (*n* = 10) were diluted 1:100 in blocking solution and then added in triplicate to wells for each individual.

Wells were washed 3 times with PBS/0.05% Tween 20 after 2 h incubation, incubated with HRP-conjugated goat anti-human IgG (1:5,000) for 2 h, washed 3 times with PBS/0.05% Tween and washed once more with PBS. Seroreactivity was detected using TMB substrate and measuring absorbance by MultiSkan Go spectrophotometer at 620 nm (Thermo Scientific). The results were expressed as reactivity indexes (RI), representing the mean optical density (OD) of each tested sample divided by the mean OD of 10 malaria-naive individuals' samples plus three standard deviations (SD). Individuals were scored as responders to *Pv*CelTOS if the RI against the recombinant protein was higher than 1. The Mann-Whitney test was used for comparing both groups. SPSS v.20 software was used for all analysis.

### Fluorescence Activated Cell-Sorting Experiments

HepG2 (hepatoma) and HeLa (epithelial) cells at 70–80% confluence were detached, washed in PBS and percentage cell viability checked. Total binding involved 1 × 10^6^ HepG2 or HeLa cells being incubated overnight at 4°C with 25 μg r*Pv*CelTOS; 1 × 10^6^ HeLa cells were incubated with 25 μg r*Pv*CelTOS in the absence or presence of each r*Pv*CelTOS-derived synthetic peptide (>99% purity) in a 1:100 (protein-peptide) molar ratio for the competition assay. Unbound protein was removed by spinning; cells were then washed with 1% BSA/PBS and incubated with an anti-histidine tag allophycocyanin (APC)-conjugated Ab (R&D Systems) for 20 min at room temperature (RT) following incubation with propidium iodide (PI) (Thermo Fisher) as viability marker. The amount of stained cells was read on a FACSCanto II (BD Bioscience) and the results were analyzed using FlowJo software (Tree Star). Stained cells without recombinant protein were used as control in these experiments. r*Pv*CelTOS binding in the absence of peptides was considered 100% binding in competition assays.

### Radiolabelling r*Pv*CelTOS and Binding Assays

Fifteen microgram soluble r*Pv*CelTOS was radiolabelled with 4 μL Na^125^I (100 mCi/mL; ARC) and iodination beads (Pierce-Thermo Scientific), following the manufacturer's instructions. Following 15 min incubation, radiolabelled recombinant proteins were separated by size-exclusion chromatography on a Sephadex G-25 column (Pharmacia). Each eluted fraction was then analyzed by gamma counter (Packard Cobra II).

Binding assays involved incubating 1.2 × 10^6^ HeLa or HepG2 cells with different concentrations of radiolabelled recombinant protein (0–300 nM) at RT for 90 min in the absence (total binding) or presence (non-specific binding) of the same unlabelled recombinant protein (2 mM). HeLa cells were pre-treated with 500 μU/mL heparinase I (CAS; 9025-39-2, Sigma), heparinase II (CAS; 149371-12-0, Sigma), chondroitinase ABC (CAS; 9024-13-9), or chondroitinase AC (CAS; 9047-57-8) for 2 h at 37°C to assess receptor susceptibility to enzymatic treatment. The cells were then washed and assessed in a conventional binding assay, using untreated cells as positive control. The mixture (cell-recombinant protein) was passed through a 60:40 dioctylphthalate-dibutylphthalate cushion and spun at 4,500 × g for 5 min. Cell-associated radioactivity was quantified on a gamma counter.

### Immunofluorescence Assays

HeLa cells (30,000 per slide) were grown on coverslips, then washed in phosphate buffered saline (PBS), fixed with 2% formaldehyde (in PBS) for 15 min at RT and blocked with 10% fetal calf serum in PBS for 1 h. The cells were then incubated with 25 μg *Pv*CelTOS in the absence or presence of 40833, 40835, or 40838 synthetic peptide (>99% purity) in a 1:100 (protein-peptide) molar ratio for the competition assay at 4°C overnight. The coverslips were washed twice with PBS and then incubated with anti-histidine monoclonal Ab (mAb) (Sigma) at 1:4,000 dilution for 1 h at RT. Following two washes with PBS, the cells were incubated with monoclonal FITC-conjugated anti-mouse secondary Ab. Cell nuclei were stained with 4′,6-diamidino-2-phenylindole (DAPI) and fluorescence was visualized by fluorescence microscope (Olympus BX51) using an Olympus DP2 camera and Fiji software. HeLa cells without protein or with histidine peptide (HHHHHH) were incubated with primary and secondary Abs as control.

### *Pv*CelTOS 3D Model

*Pv*CelTOS crystal structure (PDB:5TSZ) (Jimah et al., 2016) was modeled using VMD 1.9.3 (Humphrey et al., 1996). The peptides being analyzed were depicted in the structure and POLYVIEW software (Porollo et al., 2004) was used for calculating relative solvent accessibility, aa physical-chemical profile and secondary structure annotation. The BepiPred server was used for predicting B-cell epitopes (at 0.5 default and 75% specificity) (Larsen et al., 2006). The NetMHCIIpan-3.2 server was used for predicting T-cell epitopes from selected regions (Jensen et al., 2018).

## Results

### *pvceltos* Genetic Diversity

Bioinformatics analysis has already shown that *Pv*CelTOS is a 21.3 kDa (CDS: 591 bp) secretory protein (signal peptide between positions 1–32) lacking transmembrane helices, GPI anchors or defined domains (Garzon-Ospina et al., 2018). A 546 bp fragment (the region encoding the signal peptide was excluded) was amplified from 62 samples of parasite DNA obtained from two endemic areas of Colombia. All the sequences had the same length, thereby discarding length polymorphism.

The 62 Colombian sequences were compared to sequences obtained from regions worldwide (Carlton et al., 2008; Neafsey et al., 2012; Auburn et al., 2016; Mehrizi et al., 2017). Analyzing 209 sequences showed that *pvceltos* is highly conserved worldwide (Table 1). This gene had just seven segregating sites in the *P. vivax* population worldwide. Three of them were found as singleton sites and thus considered rare SNPs or low frequency SNPs. The remaining segregating sites (parsimonious sites) were shared amongst worldwide populations.

**Table 1:** Estimators for predicting *pvceltos* genetic diversity in Colombia and other regions worldwide.

***n***	**Sites**	**Ss**	**S**	**Ps**	**H**	**θ^w^ (SD)**	**π (SD)**
**Worldwide isolates**							
209	546	7	3	4	11	0.00217 (0.00082)	0.00184 (0.00013)
**Colombian isolates**							
85	546	2	1	1	4	0.00073 (0.00052)	0.00084 (0.00009)
**Choco isolates—Colombia**							
46	546	1	0	1	2	0.00042 (0.00042)	0.00092 (0.00042)
**Cordoba isolates—Colombia**							
16	546	1	1	0	2	0.00055 (0.00055)	0.00023 (0.00019)
**Meta isolates—Colombia**							
23	588	2	1	1	4	0.00092 (0.00065)	0.00069 (0.00023)
**Mexican isolates**							
15	588	2	2	0	3	0.00105 (0.00074)	0.00045 (0.00026)
**Peruvian isolates**							
24	588	1	1	0	2	0.00046 (0.00046)	0.00014 (0.00013)
**Brazilian isolates**							
4	588	2	1	1	3	0.00186 (0.00131)	0.00198 (0.00062)
**Chinese isolates**							
6	588	3	2	1	5	0.00223 (0.00129)	0.00249 (0.00054)
**Thai isolates**							
12	588	4	2	2	5	0.00225 (0.00113)	0.00260 (0.00048)
**Iranian isolates**							
46	588	3	0	3	5	0.00116 (0.00067)	0.00169 (0.00116)
**Indian isolates**							
2	588	2	2	0	2	0.00340 (0.00241)	0.00340 (0.00170)
**Cambodian isolates**							
3	588	1	1	0	2	0.00113 (0.00113)	0.00113 (0.00053)
**Papua New Guinea**							
5	588	2	2	0	3	0.00163 (0.00115)	0.00136 (0.00051)
**Madagascan isolates**							
3	588	1	1	0	2	0.00113 (0.00113)	0.00113 (0.00113)

*Genetic diversity estimators were calculated for the 209 sequences available in databases (worldwide isolates) and by their place of origin. Colombian sequences included the 62 sequences obtained in this study (from Colombia's Choco and Cordoba departments), together with 23 sequences available in PlasmoDB (from the Meta department). n, amount of isolates; sites, total of sites analyzed excluding gaps; Ss, amount of segregating sites; S, amount of singleton sites; Ps, amount of informative-parsimonious sites; H, amount of haplotypes; θw, Watterson estimator; π, nucleotide diversity per site; SD, standard deviation. The genetic diversity data from the small amount of isolates is only informative; it should therefore be treated with caution*.

There were just two segregating sites in the Colombian parasite population. One (identified as a parsimonious site) was found from the sequences reported here (GenBank access numbers MK913678–MK913739) and the other (position 352 according to Sal-I numbering, a singleton site) was found in Colombian sequences available in PlasmoDB. Two SNPs were found at this site, both being non-synonymous sites.

**Figure 1:**
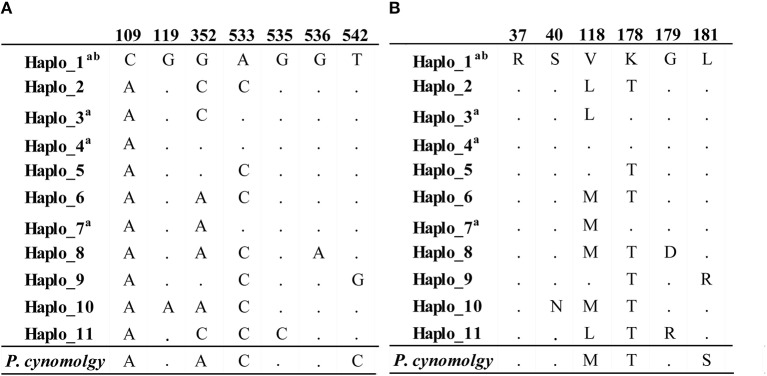
*pvceltos* haplotype (allele) alignment. The alignment shows the 11 DNA haplotypes **(A)** and the 10 encoded aa sequences **(B)** found in the 209 sequences analyzed here, together with the *P. cynomolgi* haplotype (bottom). The numbers at the top indicate the nucleotide and aa position where segregating sites or aa changes were observed. The numbering is based on the Sal-I strain sequence. ^a^Haplotypes found in Colombia; ^b^Sal-I strain haplotype. The dots indicate nucleotide or aa identity. Haplotype 1 and 4 encoded the same aa sequences. The alignment shows the positions where polymorphism was observed.

Worldwide nucleotide diversity (π) was 0.00217 and Watterson estimator (θ^W^) was 0.00184. The π value was 0.0008 in Colombia; this value was greater than that observed in Mexico and Peru (Table 1) but lower than that for Iran (π = 0.001) and other Asian countries (Table 1). Eleven haplotypes (alleles) were observed in 209 DNA sequences circulating worldwide, encoding for 10 aa sequences (Figure 1). Four DNA haplotypes were observed (Figure 1, Haplo_1, _3, _4, and _7), encoding three aa sequences in Colombia.

Neutral theory-based tests (Kimura, 1983) were used for testing the null hypothesis regarding molecular evolution (segregating sites are selectively neutral), thereby determining the main evolutionary forces that produced the diversity pattern observed in *pvceltos*. No significant values were observed regarding the Tajima or Fu and Li estimators (Table 2). However, statistically significant negative H values were observed in the Fay and Wu test for the Colombian and Mexican populations (Table 2), negative values for this test being observed in the region encoding the *Pv*CelTOS C-terminal portion (Supplementary Material 1).

**Table 2:** Tests based on the neutral model of molecular evolution.

***n***	**Population**	**Tajima**	**Fu & Li**		**Fay & Wu's H (*p*-value)**	**Fu's Fs (*p*-value)**
		**D (*p*-value)**	D* (*p*-value)	F* (*p*-value)		
85	Colombia	−0.43724 (>0.1)	−0.56533 (>0.1)	−0.61662 (>0.1)	*−1.6112 (<0.05)*	−0.705 (>0.1)
46	Iran	0.96472 (>0.1)	0.75207 (>0.1)	0.81904 (>0.1)	−0.22029 (>0.1)	−0.207 (>0.1)
24	Peru	−1.15933 (>0.1)	−1.65357 (>0.1)	−1.76132 (>0.1)	0.07971 (>0.1)	−1.028 (>0.1)
15	Mexico	−1.49051 (>0.1)	−0.56475 (>0.1)	−0.93440 (>0.1)	*−1.60952 (<0.05)*	−1.546 (<0.05)
12	Thailand	−0.27918 (>0.1)	0.30075 (>0.1)	0.29512 (>0.1)	0.18182 (>0.1)	−0.855 (>0.1)

n, amount of isolates used. Only populations having 10 or more isolates were taken into account for this analysis. Tajima's D, Fu & Li's D^*^ and F^*^, Fay & Wu's H and Fu's Fs tests were used for testing the hypothesis that all mutations were selectively neutral. Fu's and Li's D^*^ and F^*^ estimators were computed without taking an outgroup into account. Statistically significant values lower than 0 could have been due to balancing selection or genetic drift whilst values higher than 0 indicated directional selection or population expansion. Significant negative H values indicated selective sweep. Fu's Fs was considered significant only if p-values were lower than 0.02. Statistically significant negative H values observed for the Colombian and Mexican populations are shown in italics.

**Figure 2:**
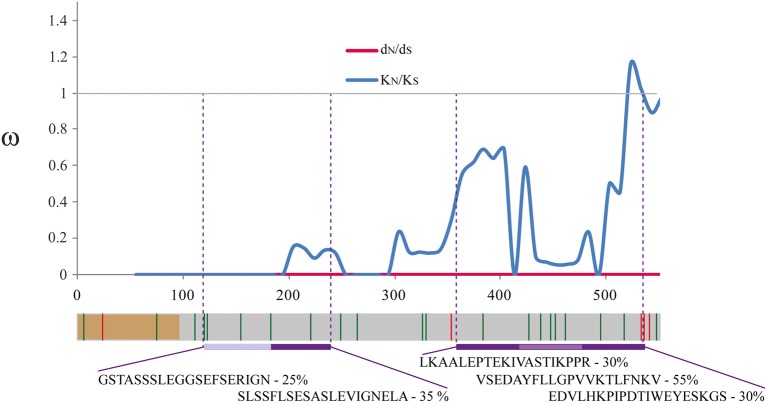
Sliding window analysis for ω rates. Natural selection signals may differ along the genome. An evolutionary omega (ω) rate sliding window was inferred to assess natural selection signatures throughout the celtos gene. The ω (dN/dS) values for *pvceltos* (using the 209 *P. vivax* sequences) are shown in red and values for divergence (ω: K_N_/K_S_) between *P. vivax* (*n* = 209) and *P. cynomolgi* (*n* = 1) are shown in blue. A gene diagram is given below the sliding window. The region encoding the signal peptide is shown in brown, positive selection sites are highlighted by red lines whilst negative selection sites are depicted with green lines. Peptide sequences having recombinant protein binding inhibition are shown in purple. Regions having an ω higher than 1 are expected to be under positive selection whilst regions with an ω lower than 1 are those expected to be under negative selection. Regions under functional constraint are expected to display a negative selection signature (ω < 1, codons under negative selection), being highly conserved between closely related species.

The d_N_-d_S_ difference was not statistically significant (d_N_-d_S_ = −0.000036, *p* > 0.05); likewise, codon-based methods revealed no evidence of positively or negatively selected codons within *P. vivax*. No significant differences were observed between polymorphism and divergence using the MK test. The difference between non-synonymous and synonymous divergence rates was not statistically significant (K_N_-K_S_ = −0.000965, *p* > 0.05); however, five sites were under positive selection and 21 under negative selection amongst species (Figure 2) according to codon-based methods. The latter were concentrated where sliding window ω rate values were lower than 1 (indicator of negative selection) (Figure 2).

**Figure 3:**
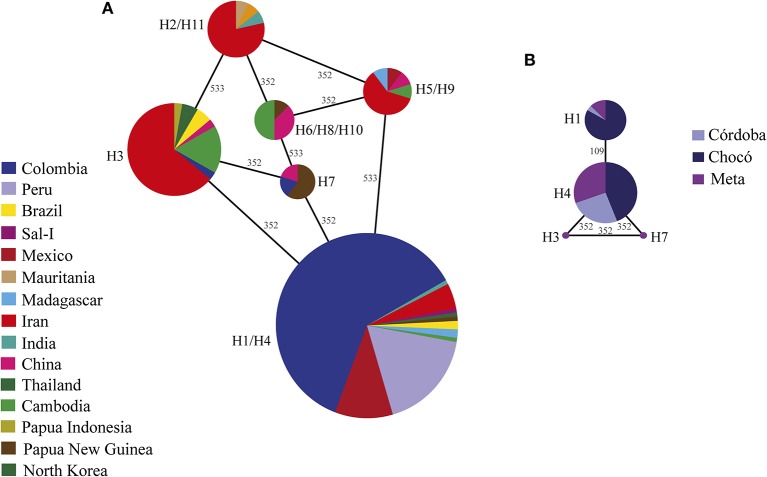
Haplotype networks. A haplotype network was created by the Median-Joining method using the 209 available sequences **(A)** and another for just Colombian sequences (*n* = 85) **(B)** to represent the relationships amongst the different haploid genotypes observed in the dataset. A haplotypes H1 and H4 were contracted within the same haplotype (H1/H4, they had the same aa sequences) whilst the haplotypes represented by just one sequence (H6, H8, and H10) were contracted together with haplotypes having high frequency and similar sequences. Circle size represents haplotype frequency whilst different colors indicate the sequences' geographical origin. The branches (lines) connecting haplotypes represent the mutational steps where the numbers on branches indicate the position where a nucleotide became changed for another one.

A haplotype (allele) network was then constructed involving all the available sequences (Figure 3A) and again with just the Colombian sequences (Figure 3B). The 209-sequence haplotype network was similar to that reported previously (Mehrizi et al., 2017), even though mutational paths were less complex and the network was easier to interpret. Six main haplotypes were identified, all connected by a single mutational step (Figure 3A). The American sequences (from Colombia, Mexico, Peru, Brazil, and Sal-I) were mainly located in haplotype H1/H4 in which some Asian and African sequences were observed. The sequences from Asian countries were located in the other five haplotypes, American sequences being less representative (Figure 3A).

A genetic difference was observed between Asia and America when calculating the fixation index (F_ST_, Supplementary Material 2), which was to be expected and coincided with the haplotype network. The sequences from the three Colombian subpopulations (Chocó, Córdoba, and Meta) were mainly grouped in haplotypes H1 and H4, these being connected by a single mutational step (Figure 2). Haplotypes H3 and H7 occurred infrequently and were apparently exclusive to the Meta region. The F_ST_ values were significant concerning the Chocó region and the other two Colombian subpopulations, but not between Córdoba and Meta (Supplementary Material 2). No evidence of genetic recombination was found; therefore, this evolutionary force would not be involved in producing new allele variants.

### Measuring r*Pv*CelTOS Antigenicity

**Figure 4:**
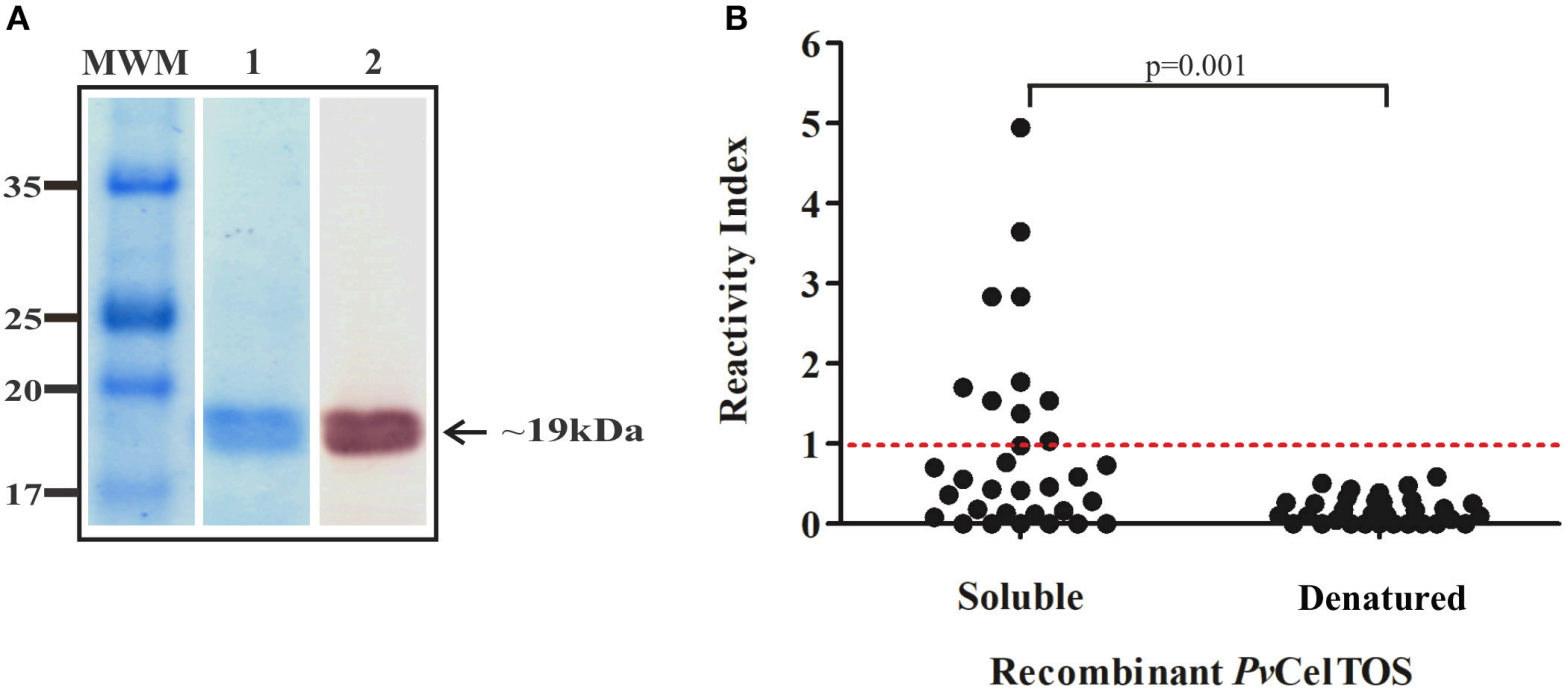
Soluble recombinant *Pv*CelTOS was recognized by *P. vivax* infected individuals' sera. Full-length recombinant *Pv*CelTOS protein was obtained in *E. coli* as a soluble protein and purified by affinity chromatography. A band of about 19 kDa was observed by Coomassie staining (1) and Western blot (2). MWM, molecular weight marker **(A)**. Soluble r*Pv*CelTOS was recognized by sera from *P. vivax*-infected individuals. The figure shows specific IgG Ab reactivity indices. The red line represents the cutoff reactivity index (RI) considered for classifying individuals as positive (>1) or negative (<1) for *Pv*CelTOS **(B)**. Analysis was carried out in triplicate. The Mann-Whitney test was used for comparing both groups.

IgG reactivity against recombinant antigen was determined to ascertain whether *Pv*CelTOS could induce a naturally-acquired humoral response in Colombian individuals from endemic regions. *Pv*CelTOS was produced in *E. coli* as a ~19 kDa soluble polypeptide and purified by affinity chromatography (Figure 4A). Around 30% (9/32) of the 32 sera from *P. vivax* infected patients had specific Abs against r*Pv*CelTOS (Figure 4B). Responders' IR values ranged from 1.37 to 4.94 (mean = 2.46 ± 0.65).

The recombinant protein was denatured at 80°C for 30 min and then incubated with sera from *P. vivax* infected patients to explore whether r*Pv*CelTOS had been properly folded (Figure 4B). Such treatment led to none of the sera being able to recognize denatured r*Pv*CelTOS, having 0.04–0.058 IR (mean = 0.25 ± 0.15). Statistically significant differences were found when comparing the soluble protein group to that having denatured protein (*p* = 0.001).

### r*Pv*CelTOS Bound Specifically to HeLa Cell Surface

**Figure 5:**
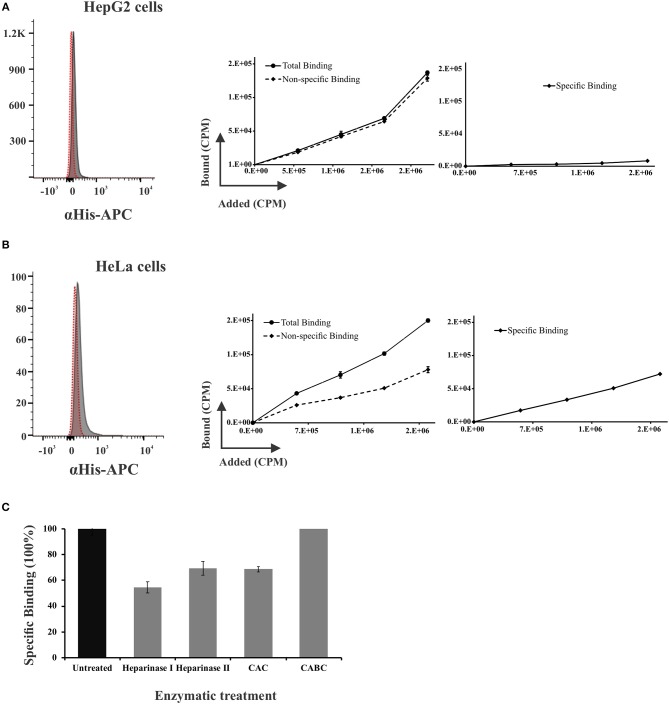
*Pv*CelTOS specific binding to host cells. *Pv*CelTOS was incubated with HepG2 **(A)** or HeLa cells **(B)**. The protein's total binding was detected by flow cytometry using anti-histidine mAb. r*Pv*CelTOS was radiolabelled and incubated with cells in the absence (total binding) or presence (non-specific binding) of non-radiolabelled protein (right-hand panel) for measuring its specific binding. The specific binding curve was calculated by subtracting total binding from non-specific binding. r*Pv*Celtos binding to HeLa cells pre-treated with enzymes cleaving glycosaminoglycans. r*Pv*Celtos binding to untreated HeLa cells was considered as 100% binding. Standard deviations were below 5%. **(C)**. Flow cytometry was used for measuring total binding, with three lots of recombinant protein. The most representative experiment is shown. Radiolabelling assays were carried out in triplicate in two independent experiments.

Flow cytometry-based assays were used for analyzing soluble r*Pv*CelTOS ability to bind to HepG2 or HeLa cells. Figure 5 shows that a shift in histogram signal (gray) was only observed in the presence of recombinant protein (for both types of cell); total binding to HeLa cells was 14% ± 2.5 and 18.5% ± 5 to HepG2 cells. Total binding was measured by flow cytometry with three lots of recombinant protein.

The recombinant protein was then radiolabelled and incubated with each cell type in the absence (total binding) or presence (unspecific binding) of unlabelled protein to evaluate whether such interaction was specific (Figure 5). The amount of radioactive protein bound in the absence of unlabelled protein was referred to as total binding. To distinguish binding to specific sites from binding to non-specific sites, a second set of incubations was run simultaneously using radioactive protein and the same protein which was not radiolabelled at a concentration sufficient to block radioactive protein binding to the specific sites, but not non-specific sites. Specific binding was calculated using such data by subtracting unspecific binding from total binding. The binding graphs showed that *Pv*CelTOS had specific HeLa binding (3% ± 0.1) compared to low HepG2 binding (0.3% ± 0.5) (Figure 5).

HeLa cells were pre-treated with glycosaminoglycans, heparin lyases, chondroitinase ABC, and chondroitinase AC to determine whether *Pv*CelTOS binding to HeLa cell membrane depended on the presence of proteoglycans (involved in some proteins' binding, i.e., CSP and TRAP). Figure 5C shows that no enzyme could abolish *Pv*CelTOS binding: however, heparinase 1 reduced recombinant proteins' specific binding by 45% whilst heparinase II and chondroitinase AC achieved a 31% reduction. Radiolabelling assays were carried out in triplicate in two independent experiments. The SD were lower than 5% for enzyme treatments.

**Figure 6:**
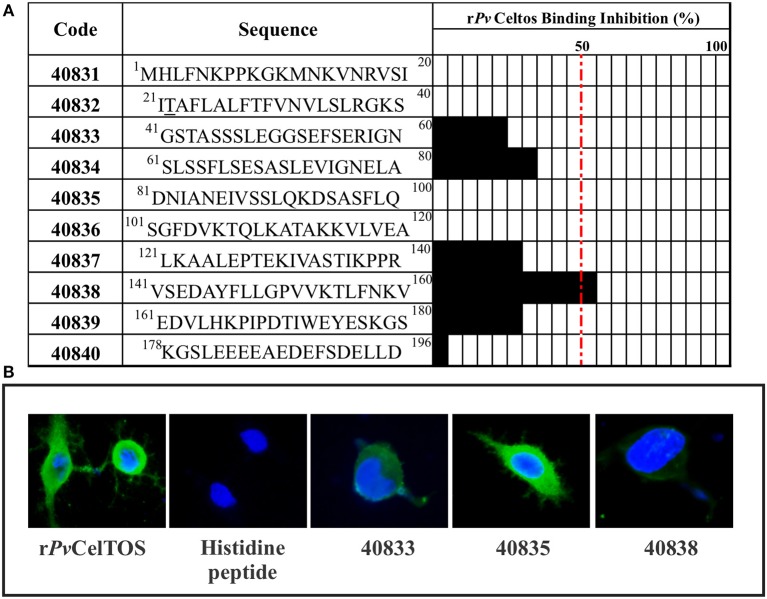
*Pv*CelTOS competition assays. Twenty amino acid-long, non-overlapping peptides covered the length of *Pv*CelTOS were used in the r*Pv*CelTOS HeLa cell binding inhibition assays. The black bars represent the percentage regarding the extent to which each peptide inhibited recombinant protein binding. Peptides which were able to inhibit r*Pv*CelTOS binding coincided with regions where sites under negative selection were observed. Each assay was carried out in triplicate in two independent experiments. Standard deviations were below 5% **(A)**. Competition assays using immunofluorescence. The two most representative peptides (40833 and 40838) were able to reduce *Pv*CelTOS binding compared to just the protein or in the presence of peptide 40835 **(B)**. r*Pv*CelTOS binding to HeLa cell membrane is shown as a green fluorescence pattern whilst blue shows cell nuclei. Competition assays were carried out in triplicate.

**Figure 7:**
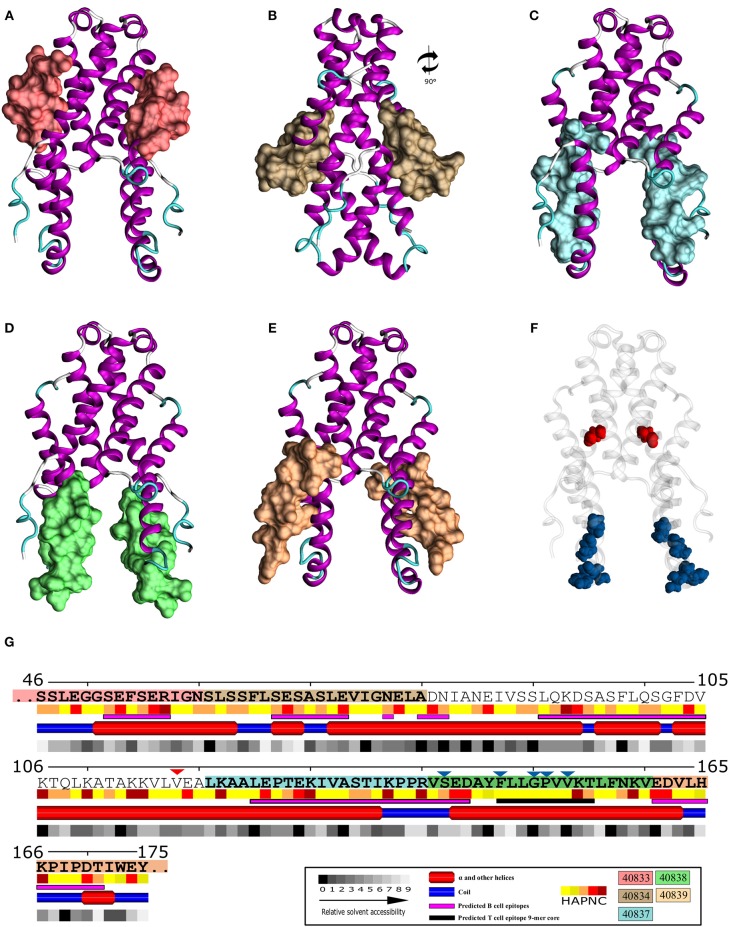
*Pv*Celtos protein structure model. Location of the studied peptides in *Pv*CelTOS dimer structure (Jimah et al., 2016): **(A)** Peptide 40833 (red), **(B)** Peptide 40834 (brown), **(C)** Peptide 40837 (light blue), **(D)** Peptide 40838 (green), **(E)** Peptide 40839 (orange). **(F)** Positions submitted to selective pressure; positive selected positions: 118 (red) and negative selected positions 142, 147, 150, 151, and 153 (blue). **(G)** Secondary structure representation, including relative solvent accessibility (0-completely buried, 9-fully exposed) and physical-chemical aa profile (H-hydrophobic: A,C,F,G,I,L,M,V, A-amphipathic: H,W,Y, P-polar: N,S,Q,T, and N/C-charged: D,E-neg; R,K-pos). Peptide sequences are showed in bold; positions submitted to positive selection pressure are indicated by red triangles and negatively selection ones by blue triangles. Predicted B- (purple bar) and T-cell epitopes (black bar) are also indicated.

A competition assay between r*Pv*CelTOS and non-overlapping synthetic peptides derived from the same protein was used for determining which protein region mediated binding to HeLa cell surface. Five peptides were able to specifically inhibit recombinant protein binding to target cells, peptide 40383 (located in the protein's C-terminus) being able to inhibit binding by more than 50%, followed by 40834 (35% inhibition) and 40833, 40837, and 40839 (the last three having 25% inhibition) (Figures 6A, 7A–E). Immunofluorescence assays were carried out to visualize *Pv*CelTOS binding to HeLa cell membrane. No fluorescence signal was observed when cells were incubated with histidine peptide or in the absence of protein (data not shown). Competition binding assays revealed a reduced fluorescence pattern for *Pv*CelTOS in the presence of peptide 40833 and 40838; no difference was observed in the presence of peptide 40835 (Figure 6B). The assays were carried out twice in triplicate. The SD did not exceed 5%.

### The Structural Location of *Pv*CelTOS Binding Inhibition Peptides

Each peptide capable of inhibiting recombinant protein binding to HeLa cells was localized on *Pv*CelTOS protein model (Figure 7; PDB:5TSZ, Jimah et al., 2016) to analyse its structural traits, physicochemical features and its relative solvent accessibility. In addition, the potential B-cell epitopes (BCE) and T-cell epitopes (TCE) were calculated. It was found that all peptides were solvent accessible (Figure 7G) and those located at the amino terminal extreme (40833—Figure 7A and 40834—Figure 7B) had greater accessibility to the solvent than peptides located at the carboxyl-terminal extreme (40837—Figure 7C, 40838—Figure 7D, 40839—Figure 7E). According to this characteristic, all peptides included regions with viable BCE, highlighting their potential capacity as immunogens (Figure 7G).

Overall, the most interesting region was located on α-helix four in a hydrophobic cleft (peptides 40837 and 40838), where positions under selective negative pressure were found (142, 147, 150, 151, and 153, Figures 7F,G), that were overlapping with a viable TCE. This region also contained a BCE involving the last four residues of peptide 40837 (^137^KPPR^140^) and first four residues of peptide 40838 (^141^VSED^144^), being part of a highly hydrophilic loop that could be accessible to Abs (Figure 7G).

## Discussion

The *Plasmodium* spp. life-cycle alternates between a definitive invertebrate host and human beings as intermediate host. The parasite's entry to humans involves its release in Spz form into the dermis during the vector's bite. Gliding motility and cell traversal are then activated to enable the parasites to overcome cellular barriers to cross/traverse different types of cell [dermal fibroblasts, blood vessels' endothelial cells, phagocytes in sinusoidal and dermal layers (Kupffer cells), and hepatocytes] along their route to the hepatocytes in which they will reside and develop into liver-stage parasites (Vanderberg et al., 1990; Mota et al., 2001; Amino et al., 2008).

Unlike invasion, which is obligatory for PVM formation, Spz cell traversal constitutes a transient interaction with a host cell. This has been widely studied in *Plasmodium* species affecting rodents, such as *P. berghei* and *P. yoelii*, showing how Spz breach cell plasma membrane, glide through the cytosol and exit host cells (Mota et al., 2001). However, recent studies by Risco-Castillo et al., have shown that Spz traverse hepatocytes by tight junctions within transient vacuoles (Risco-Castillo et al., 2015). Although this process is controversial, it appears to be orchestrated by a significant amount of parasite antigens, such as PLP1 (Ishino et al., 2004), SPECT (Kaiser et al., 2004), TRAP-like protein (TLP) (Moreira et al., 2008), and CelTOS (Kariu et al., 2006), and receptors on target cells, such as CD68 (found exclusively on Kupffer cells) (Cha et al., 2015) and a protein on hepatocytes known as Ephrin A2 (Kaushansky et al., 2015).

Traversal is crucial for initiating the hepatic phase; parasite proteins involved in it represent the main targets to be considered when designing a fully effective antimalarial vaccine. Therefore, for an antigen to be considered a vaccine candidate it must participate in and be critical for parasite entry to cells, be accessible to a host's immune system, have low genetic variability and trigger protection-inducing immune responses. This study has shown that 30% of *P. vivax*-infected patients' sera was able to recognize the *Pv*CelTOS soluble recombinant protein, suggesting that this antigen is exposed during natural infection. These results coincided with those from previous studies on this protein carried out in other regions where it was found that *Pv*CelTOS was naturally antigenic in Amazon region inhabitants, 94 individuals (17.8%) having specific IgG Abs against the full recombinant protein (Rodrigues-Da-Silva et al., 2017). *Pv*CelTOS antigenicity was comparable to that shown in results for other important antigens such as TRAP, which is an essential antigen for hepatocyte invasion (Muller et al., 1993).

CelTOS' immunological profile has been extensively studied by other groups in different *Plasmodium* species. Such relevant results have guided the study of CelTOS in other species, such as *P. vivax*. Immunizing *Pv*CelTOS in four clinically-relevant platforms [recombinant chimpanzee adenoviral (Ad) vector 63 (ChAd63), modified vaccinia virus Ankara (MVA) vector, virus-like particles (VLPs), and recombinant protein] has emphasized *Pv*CelTOS' high immunogenic potential, even after a single prime-boost immunization with ChA63-*Pv*CelTOS. Ab levels have increased significantly after boosting with MVA, VLPs, and proteins (Alves et al., 2017). Unfortunately, only a modest percentage of protection was achieved following challenge with chimeric *P. berghei* parasites expressing *Pv*CelTOS, even in the presence of anti-*Pv*CelTOS Abs and *Pv*CelTOS-specific CD8 T-cell responses (Alves et al., 2017).

New advances in immunological studies have shown the urgent need for rethinking the design for studying *Pv*CelTOS as a vaccine candidate. This would involve just the protein's regions having functional relevance (not the full-length protein) regarding host cell interaction, which would coincide with regions having functional constraints (low genetic diversity and negative natural selection signatures) for covering a broad spectrum of important *P. vivax* strains for avoiding the allele-specific immune responses characteristic of malaria.

*Pv*CelTOS has been seen to have low diversity in worldwide and Colombian parasite populations (Bitencourt Chaves et al., 2017; Mehrizi et al., 2017). The nucleotide diversity (0.0018 ± 0.0001) observed here was similar to that of other Spz stage antigens (Garzon-Ospina et al., 2018), such as *Pv*TRAP (π = 0.0059 ± 0.00029) (Chenet et al., 2012), and much lower than that for Mrz proteins (DBP region II π = 0.01103 ± 0.00025, AMA-1 π = 0.01653 ± 0.00016) (Chenet et al., 2012), and members of the *pvmsp7* family (π > 0.01) (Garzon-Ospina et al., 2012).

Eleven DNA haplotypes were found worldwide, of which just four were present in the Colombian parasite population. Genetic differentiation analysis revealed that a Thai/Iranian population was greatly differentiated from American populations (Peru, Mexico, and Colombia), this was to be expected due to the distance between these populations. Significant values were observed in Colombia between Chocó and the other subpopulations which could have resulted from structuration in Colombia (limited gene flow amongst populations), as described earlier for other antigens (Forero-Rodriguez et al., 2014; Buitrago et al., 2016; Camargo-Ayala et al., 2018).

The low genetic diversity observed in *pvceltos* could have resulted from functional constraint in protein regions (i.e., the amount of acceptable nucleotide/aa changes without negatively affecting gene/protein function or structure) or from selective processes which have favored a particular haplotype. Negative statistically significant H values were observed for the Fay and Wu test in Colombian and Mexican populations, suggesting a selective sweep. Such selective force reduced diversity due to a certain variant's increased frequency due to positive natural selection. A Fay and Wu test sliding window suggested that the selected variant might have been located at the gene's 3′ end. The ω rate sliding window could have confirmed this statement. The ω rate gave values >1 in the gene's 3′ end, indicating a greater accumulation of non-synonymous changes in the C-terminal region. Furthermore, codon-based tests showed that residues 118, 178 and 179 were under positive selection (Figure 2). The aa encoded by these codons (V118L, K178T, and G179R) were located in *Pv*CelTOS CD8+ T-cells' epitope regions (Mehrizi et al., 2017), suggesting that they could have increased their frequency (eliminating other variants in this position and neighboring ones) due to an adaptive advantage (positive natural selection) which would have helped in avoiding recognition by a host's immune system.

The sliding window also gave values lower than 1 (a signature for negative natural selection) throughout the sequence; ω < 0.3 values were observed toward the gene's 5′-end and 3′-end. Codon-based tests found negative selection signals in 21 codons; several of them were located where a ω < 0.3 was observed. Regions having negative selection signals would be expected to be under functional constraint (Kimura, 1983; Graur et al., 2013); therefore, the *Pv*CelTOS regions having a ω < 0.3 and negative selection sites could be the protein's functional regions (for instance, these regions could have been involved in parasite-host interaction).

The first task involved in confirming this hypothesis was to evaluate *Pv*CelTOS ability to bind to target cells. There is a large body of evidence supporting CelTOS participation in malaria parasites' Spz cell traversal in the mosquito vector and human host, therefore being critical for malarial transmission and disease pathogenesis in *P. berghei* and *P. yoelii* (Kariu et al., 2006; Jimah et al., 2016; Steel et al., 2018). However, it is still not clear whether its participation was mediated by specific interaction with receptors on target cells. Previous studies have shown that *Pv*CelTOS and *Pf* CelTOS specifically interacted with phosphatidic acid to enable parasite exit from cells during traversal (Jimah et al., 2016). However, this does not rule out its participation in cell entry since it seems to be able to trigger immune responses, suggesting its exposure to the immune system during cell traversal (Figure 4).

*Pv*CelTOS was obtained as a soluble protein in *E. coli* (Figure 4A); this is important, as the protein was not denatured with chaotropic agents, thereby guaranteeing that the protein maintained the protein sequence's hydrophobic interactions. Even though structural studies have shown that *Pv*CelTOS does not contain disulphide bonds or high complexity structural elements for obtaining it or its stability (Jimah et al., 2016), this study found that natural Ab recognition became significantly reduced when soluble protein produced in *E. coli* was submitted to high temperatures (Figure 4B). This suggested that *Pv*CelTOS contained conformation-sensitive epitopes and that the recombinant protein was properly folded.

Soluble *Pv*CelTOS was then tested in a highly sensitive, robust and specific host cell-binding assay. Two types of cell were selected: HeLa (cell traversal model) and HepG2 (invasion model) cells; although HeLa cells are not involved in *Plasmodium* Spz cell traversal of the physiological epithelium, other researchers have shown wild type Spz ability to traverse such cells by cell wound assay (Ishino et al., 2004; Kariu et al., 2006). This would suggest that HeLa cells have the necessary receptors (proteins, carbohydrates, or lipids) for proteins such as CelTOS and SPECT which are essential for cell traversal and shows that the receptors on HeLa cells are also in the epithelium (i.e., the target for Spz traversal).

Binding assays revealed that *Pv*CelTOS had similar binding percentages for HeLa (14%) and HepG2 cells (18%) (Figure 5). However, as total binding may contain non-specific binding (implying that binding to receptors was not the ligands' objective), radiolabelled *Pv*CelTOS was incubated with a high concentration of non-radiolabelled protein as competitor agent. r*Pv*CelTOS bound specifically to a receptor on HeLa cells which is probably lacking on HepG2 cells since only 0.3% ± 0.5 interaction with HepG2 cells was found when calculating its specific binding. These results provided greater certainty that protein interaction with HeLa cell membrane surface is a specific interaction and supported competition studies with *Pv*CelTOS-derived synthetic peptides (Figure 5), showing how some of them could inhibit protein binding to a HeLa cell receptor in a high molar ratio. However, such results do not exclude the possibility that *Pv*CelTOS specifically interacts with other cell types.

Interestingly, no specific interaction with HepG2 cells was found; these are traversed by Spz before establishing a productive hepatocyte infection (Mota et al., 2001). It is still not clear whether the molecular basis for hepatocyte traversal is similar to that for other types of cell. It has been found that tetraspanin CD81 in hepatic cells is linked to Spz entry to hepatocytes by forming a parasitophorous vacuole but not for cell traversal since host cell traversal also occurs in CD81-deficient mice (Silvie et al., 2003). A receptor for hepatocyte invasion by multiple *Plasmodium* species has been identified recently but its role in traversal has not been examined (supposing that it has one) (Kaushansky et al., 2015). Although cell traversal seems to lack specificity regarding the type of cell to be traversed (Frevert et al., 2005; Amino et al., 2008), it has been found that a peptide called phage-display library-derived P39 specifically binds to CD68 (a major Kupffer cell surface protein), highlighting its involvement in liver infection and making this a candidate receptor for Spz traversal of Kupffer cells.

Interestingly, despite previous reports showing that the parasite can traverse hepatic cells [only with low-sulphated heparin sulfate proteoglycans (HSPGs)], P39 did not bind to hepatocytes whereas binding to macrophage-like cells (Kupffer cells and THP-1) was strong. In agreement with binding results, P39 significantly inhibited rodent *P. berghei*, and human *Plasmodium falciparum* Spz entry to macrophage-like cells but not to primary rat hepatocytes or HepG2 human hepatoma cells (Cha et al., 2015). It has been shown recently that glyceraldehyde 3-phosphate dehydrogenase (GAPDH) on parasite surface interacts with CD68 and that such interaction is critical for Kupffer cell traversal and liver infection (Cha et al., 2016). These results highlighted both specificities (cell type and type of interaction) and showed that the parasite could use different proteins for traversal according to the cell type to be traversed, making such process cell-specific.

Competition assays involved recombinant soluble proteins and 20 aa-long peptides covering the protein's entire sequence (once full-length r*Pv*CelTOS target cell binding had been confirmed) to determine whether functionally constrained regions were responsible for such binding. Interestingly, C-terminal region peptide 40838 (^141^**VSEDAYFLLGPVVKTLFNKV**^160^) was able to inhibit full-length protein binding by more than 50%, suggesting that it forms part of the region involved in host-pathogen interaction. This peptide is encoded by the gene's 3′-end which had ω < 0.3 and several sites under negative selection. Five of this peptide's aa (142, 147, 150, 151, and 153) had negative selection signals (Figures 7F,G), supporting the idea of functional constraint on this region, similar to the described previously (Garzon-Ospina et al., 2018).

Interestingly, part of this peptide shared 13 residues PTEKIVASTIKPPR**VSEDAYFLLGPVV** (letters in bold) with the epitope having the highest recognition by sera from naturally-infected *P. vivax* patients (Rodrigues-Da-Silva et al., 2017). It was found that both sequences (peptide 40838 and the epitope) were located in a hydrophobic cleft, as seen in the previously reported *Pv*CelTOS 3D structure (Jimah et al., 2016; Figure 7D). Despite being located in a hydrophobic cleft, *in silico* prediction (Figure 7G) and previous experimental results (Rodrigues-Da-Silva et al., 2017) have shown this epitope (including 13 residues from peptide 40838) to be a good B-cell epitope.

This region's solvent accessibility and physical-chemical profiles suggested that part of the epitope, including the first four residues of peptide 40838 (^141^VSED^143^) (Figure 7G), belonged to a highly hydrophilic disordered region that could be accessible to Abs. By contrast, residues ^144^AYFLLGPVVKTLF^156^ belonged to the most hydrophobic region found in all the protein, having several aa under negative selective pressure (Figures 7D,F,G). Interestingly, T-cell epitope prediction for this region showed that ^147^FLLGPVVKT^154^ residues could be presented by DRB1_0101 (score: 6.0), DRB1_1001 (score: 10), DRB1_1601 (score:9.5), and DRB1_1602 alleles (score:6.5) having significant predicted binding scores. This analysis showing contiguous B- and T-cell epitopes, as well as the ability to inhibit recombinant *Pv*CelTOS binding (showed the highest binding inhibition %), has highlighted this peptide as an interesting candidate for designing control methods against *P. vivax*. Future work should evaluate naturally-acquired Abs' capability for blocking *Pv*CelTOS binding to HeLa cells and correlate the results with some degree of protection.

Since the binding of other Spz proteins (such as CSP and TRAP) to host cells has become reduced by enzyme treatment involving cleaving glycosaminoglycans (Muller et al., 1993; Rathore et al., 2002), a similar approach was followed here. *Pv*CelTOS binding to HeLa cells remained above 50% after enzyme treatment, as reported for *Pf* CelTOS where treatment with heparinase I, heparinase II, chondroitinase CABC, and chondroitinase AC did not significantly affect binding (Curtidor et al., 2012). This suggested that the *Pv*CelTOS receptor has a protein or lipid fragment that was not considered in the experiment mentioned above. Additional studies are thus necessary to establish the receptor's identity.

Amino-terminus peptides 40833 and 40834 and carboxyl-terminus peptides 40837 and 40839 were able to inhibit protein binding by ~20–30%. It should be stressed that peptide 40833 (residues 41–80) had a completely conserved sequence compared to isolates from endemic regions worldwide and had functional constraint signals, such as being conserved amongst species having negative natural selection signatures (regions having ω < 0.3 showing sites under negative selection). The combination of bioinformatics data and experimental assays led to proposing peptides 40833 and 40838 as promising candidates for inclusion in a peptide-based, multi-antigen, multistage anti-*P. vivax* vaccine.

## Conclusions

Similar to that reported regarding other populations, *Pv*CelTOS was seen to be highly conserved and antigenic (Longley et al., 2016; Rodrigues-Da-Silva et al., 2017) only 11 haplotypes (alleles) were found in more than 200 sequences analyzed from different parts of the world. Although CelTOS has been described as a protein enabling parasite exit from host cells, our results suggested that it is also involved in the initial recognition of host cells during cell traversal.

One region in the *Pv*CelTOS amino-terminal region and another in its C-terminus were seen to be participating in host-pathogen interactions. These regions had important functional constraint signals (ω < 0.3 and several sites under negative selection) and were able to inhibit r*Pv*CelTOS' specific binding to HeLa cells. This led to suggesting that sequences between aa 41-60 (40833) and 141–160 (40838) represented promising candidates for a subunit-based vaccine against *P. vivax* since they met 3 out of 4 vaccine candidate criteria (being involved in invasion, exposed to the immune system and lacking polymorphism). Future studies in animal models are needed to further evaluate these regions' protection-inducing ability.

## Data Availability Statement

The datasets generated for this study can be found in the GenBank NIH database, using the following accession numbers: MK913678–MK913739.

## Ethics Statement

The studies involving human participants and the procedures involved in sample taking were reviewed and had been approved by Fundación Instituto de Inmunología de Colombia (FIDIC) (IRB: ACTA N° 037-CEEPA) and the Universidad del Rosario's ethics committees (IRB: CEI-ABN026-0001061). The patients/participants provided their written informed consent to participate in this study.

## Author Contributions

GA-P devised and designed the study, performed r*Pv*CelTOS radiolabelling, binding assays, and wrote the manuscript. DG-O performed the molecular evolutionary analysis and wrote the manuscript. FP expressed the recombinant proteins and carried out the antigenicity studies. MB performed the flow cytometry experiments, r*Pv*CelTOS radiolabelling, and binding assays. JF-R devised the study, performed the molecular evolutionary analysis, and wrote the manuscript. XR-M expressed the recombinant proteins and carried out the binding assays. LR-G performed the molecular evolutionary analysis. CS carried out the *in silico* predictions and prepared the 3D structure analysis. MP wrote the manuscript and coordinated the study. All authors have read and approved the final version of the manuscript.

### Conflict of Interest

The authors declare that the research was conducted in the absence of any commercial or financial relationships that could be construed as a potential conflict of interest.
