# Supplementary material for: Plasmodium vivax Cell Traversal Protein for Ookinetes and Sporozoites (CelTOS) Functionally Restricted Regions Are Involved in Specific Host-Pathogen Interactions
Source: Front Cell Infect Microbiol. 2020 Mar 24;10:119. doi: 10.3389/fcimb.2020.00119 (PMC7105572; doi:10.3389/fcimb.2020.00119)
Supplement: Supplementary file 1 [file Data_Sheet_1.PDF]

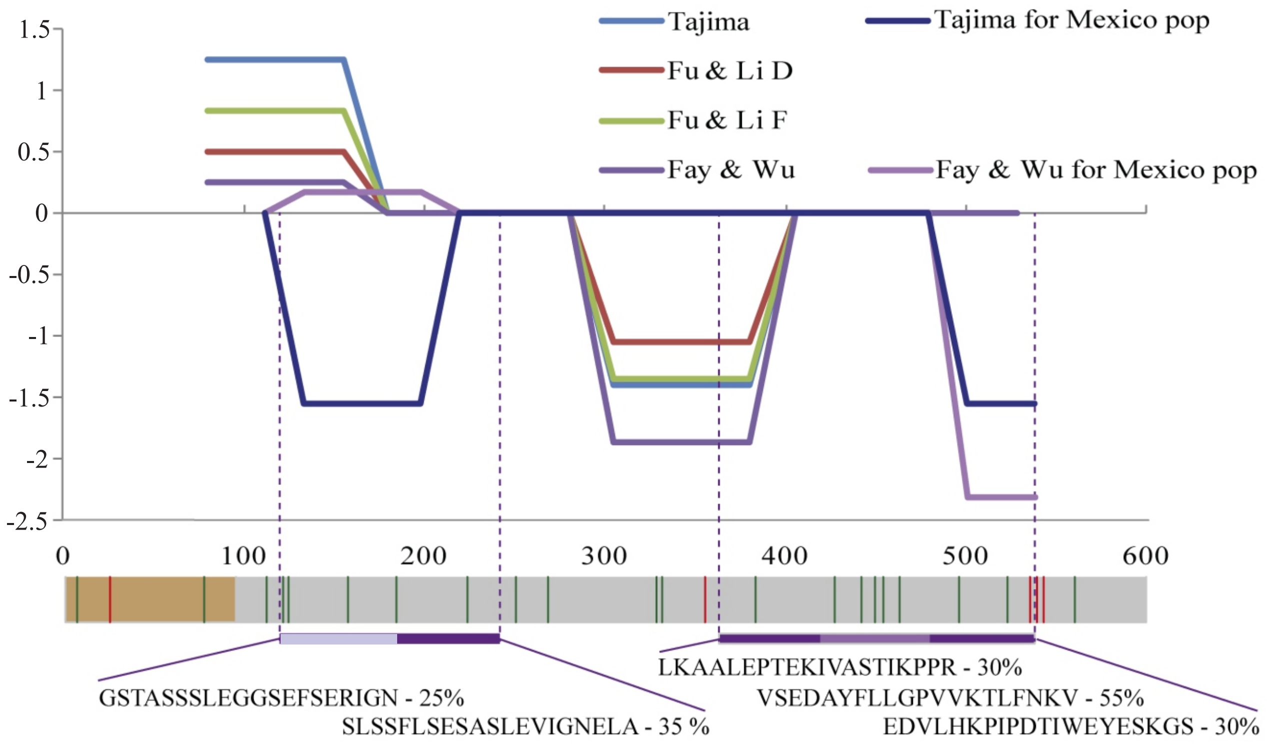

Supplementary material 1. Sliding window analysis for Tajima and Fay and Wu tests. Both the Tajima and Fu and Li tests lacked statistically significant values; however, the Fay and Wu test gave a significant value for Colombian and Mexican populations, suggesting that a selective sweep might have taken place. The region under sweep was likely to be where the negative H values were found and close to the deeper valley shown by the Tajima test. Regarding Colombia, the sweep was located around the 300 and 370 nucleotide region. The 352 SNP (codon position 118) was found in this region, which was under positive selection according to codon-based methods (FEL, REL and MEME). The sweep region for the Mexican population could have been towards 3'-end in the region enclosing the 533 SNP (codon position 178) which was found to be under positive selection by the REL method. More sequences are needed to ascertain such hypothesis. A gene diagram is shown below the sliding window. The region encoding the signal peptide is shown in brown, positive selected sites in red and negative selected sites in green. The sequences of peptides inhibiting recombinant protein binding are indicated in purple. These peptides coincided with regions where sites under negative selection were observed.

**Population**	Thailand	Peru	Mexico	Chocó	Córdoba	Meta	Iran
Thailand		0.00	0.00	0.00	0.00	0.00	0.14
Peru	**0.644**		0.56	0.00	0.99	0.13	0.00
Mexico	**0.486**	-0.009		0.01	0.73	0.67	0.00
Chocó	**0.387**	**0.284**	**0.177**		0.01	0.02	0.00
Córdoba	**0.569**	-0.050	-0.041	**0.229**		0.39	0.00
Meta	**0.423**	0.047	-0.027	**0.110**	0.005		0.00
Iran	0.038	**0.494**	**0.398**	**0.353**	**0.451**	**0.359**	

Supplementary material 2. F_{ST} statistic. The fixation index (F_{ST}) was only calculated for populations having 10 or more isolates. Statistically significant values are shown in bold. The lower diagonal gives F_{ST} values, whilst the upper diagonal indicates *p* values.
